# The relationship of coping style and social support variation to glucocorticoid metabolites in wild olive baboons (*Papio anubis*)

**DOI:** 10.1007/s10329-024-01172-2

**Published:** 2024-12-12

**Authors:** Alexander J. Pritchard, Erin R. Vogel, Rosemary A. Blersch, Ryne A. Palombit

**Affiliations:** 1 Center for Human Evolutionary Sciences, Rutgers, The State University of New Jersey, New Brunswick, NJ USA; 2 Program in Human Evolutionary Sciences, Department of Anthropology, Rutgers, The State University of New Jersey, New Brunswick, NJ USA; 3 California National Primate Research Center, University of California, Davis, CA USA

**Keywords:** Social support, Stress response system, Allostatic load, Individual differences, Coping style, Personality

## Abstract

Social support, via investment in relationships of importance with others, is often emphasized as a pathway towards mediating stress. The effectiveness of social support, however, can be altered by personality differences, but the physiological consequences of such covariation are still poorly explored. How do individual differences in the functioning of the stress response system mediate access to, and use of, social support? To examine this dynamic, we investigated glucocorticoids as a biomarker of energetic activation that may also be activated by chronic psychosocial stress. For this purpose, we studied a wild anthropoid primate, i.e. the olive baboon (*Papio anubis*), for 17 months, in Laikipia District, Kenya. We measured biomarkers of energetic activation, i.e., fecal glucocorticoid metabolites (fGCms), to address whether individual differences in stress coping and social support were associated with variation in hormone levels across a period from 2018 to 2019. We found evidence for an association between social support and fGCm concentrations. This association had a discernable interaction between sex and social support: we found a negative association in male baboons, relative to females—who did not have a pronounced effect. Our findings emphasize the importance of social support in male baboons. The cost of not having diverse bonds, has been downplayed in male baboons.

**Supplementary Information:**

The online version contains supplementary material available at 10.1007/s10329-024-01172-2.

## Introduction

Organisms react to perturbations in physiological stability, such as environmental threats or challenges, via activation of the stress response system (SRS) (McEwen and Wingfield 2003). Two primary causes of chronic stress are uncertainty and anticipation of a threat (i.e., psychological or psychosocial stressors) (Del Giudice et al. 2018; Mason 1959; Sapolsky 1994). Consistent individual differences in behavioral tendencies (e.g., personality) have been shown to influence the allostatic load (i.e., cost of chronic physiological activation (McEwen and Stellar 1993)) attributable to psychosocial stress, and stress more generally (Costantini et al. 2012; Moyers et al. 2018; Sapolsky 1994; Stephan et al. 2016; Yoneda et al. 2023).Individuals can mitigate the allostatic load of psychosocial stress by maintaining high-quality relationships that facilitate social support (Cohen and Wills 1985; Uchino et al. 1996). The effectiveness of social support in reducing the deleterious effects of psychosocial stress, however, can be influenced by consistent individual differences (as reviewed in: Swickert 2010). For instance, intrinsic individual differences in the functioning of the SRS may mediate access and use of social support (Sapolsky 1994; Seyfarth and Cheney 2013; Smith 2006). Indeed, the SRS is believed to play a central role in the formation and maintenance of human social relationships (Kornienko et al. 2020; Mercado and Hibel 2017). In summation, individual differences in response to stressors can alter the formation and maintenance of social relationships (Aplin et al. 2013; Mercado and Hibel 2017; Moyers et al. 2018; Snijders et al. 2014). Therefore, individual differences can act both directly (Costantini et al. 2012; Moyers et al. 2018; Sapolsky 1994; Stephan et al. 2016; Yoneda et al. 2023) and indirectly via social support (Sapolsky 1994; Seyfarth and Cheney 2013; Smith 2006) to influence the allostatic load of psychosocial stress.

Direct evidence among nonhuman animals for the role of consistent individual differences in altering glucocorticoid (GC) levels has been observed in captivity and, to a more limited extent, in the wild (Laudenslager et al. 2011; Suomi 1991, 1997; Wingfield and Romero 2015). For example, Sapolsky and Ray (1989) showed that male baboons who consistently initiate aggressive interactions in response to actual, rather than perceived, threats tend to have reduced GC levels. These individual differences were provisionally proposed to be similar to Type A human personality profiles (Sapolsky 2004), though the construct validity of Type A has been criticized (Friedman and Booth-Kewley 1988) and typological personality metrics have come under closer scrutiny recently (Haslam 2019). Even so, personality dimensions remain implicated with health outcomes or allostatic load in humans (Stephan et al. 2016; Strickhouser et al. 2017; Yoneda et al. 2023).

We draw upon a refined theoretic framework that characterizes consistent individual differences in the SRS along a spectrum of ‘coping styles’ (Koolhaas et al. 1999; Steimer et al. 1997), a framework that has been supported by studies on various taxa (Costantini et al. 2012; Díaz et al. 2020; Ferreira et al. 2016; Gorka et al. 2016; Øverli et al. 2007; Pritchard and Palombit 2022a; Qu et al. 2018; Silva et al. 2010; Verbeek et al. 1999). A coping style refers to a suite of tendencies in response to a stressor. Individual differences in coping style are measured using a threat or challenge paradigm to test how individuals consistently differ in their responsiveness to a stressor measured along a proactive–reactive continuum. An extreme proactive coping style is characterized by: pronounced aggression, reduced risk aversion, less behavioral flexibility, less inhibition, and less social responsiveness relative to more reactive individuals (Coppens et al. 2010; Koolhaas et al. 1999). Coping styles, thus, can be understood as measurable and consistent individual differences in the nature of the response to a stressor (Koolhaas et al. 1999, 2010), placing an interpretive emphasis on quantifying alternate solutions to a stressor.

Coping style differences are consistent across stressors, including social stressors (de Boer et al. 2017), and are measurable in primates by means of behavioral responses to controlled exposures to a stressor (e.g., Pritchard and Palombit 2022a). Personality traits might be expected to covary with coping style. That is, high scores in activity, aggression, boldness, and exploratory tendencies across situations would be associated with a proactive rather than a reactive coping style (Finkemeier et al. 2018; but see Pritchard and Palombit 2022b).

Coping styles co-vary with GC levels (Bensky et al. 2017; Costantini et al. 2012; Korte et al. 1992; Moyers et al. 2018; Silva et al. 2010). Unfortunately, it is only relatively recently that attention has focused on how coping styles and GC levels interact in a wild socially complex primate species. For example, Ferreira and colleagues (2016) reported higher fecal glucocorticoid metabolite levels (fGCm) concentrations in captive capuchin monkeys (*Sapajus libidinosus*) who scored higher on a ‘self-directed’ behavior component, analogous to a reactive coping style, relative to low-scoring individuals. Similarly, Tkaczynski and colleagues (2019) reported higher fGCm concentrations in wild Barbary macaques (*Macaca sylvanus*) who scored lower on an Excitability factor, which the authors likened to being more reactive, relative to higher scoring individuals.

Though individual differences are important, the social complexity intrinsic to many primate species elevates the importance of social support and its role in buffering against allostatic load (Cohen and Wills 1985; Crockford et al. 2008; Uchino et al. 1996; Wittig et al. 2008). Integrating personality differences in responding to stress and social behavior are key to gaining insight into the associations between these phenomena. For example, Roohafza et al. (2016) found both direct effects of personality on anxiety and depression in humans, and indirect effects mediated through social support, among other effects. Such a framework is important given coping styles can be subsumed under broader personality frameworks (e.g., the Five-Factor Model, FFM) (Finkemeier et al. 2018; Pritchard and Palombit 2022b).

Validating the construct of coping styles within major human personality frameworks is challenging, partly because the measure of coping style is constrained to a particular situation as opposed to cross-situational consistency, and partly because human conceptualizations of coping style often subsume complex cognitive or socio-cultural processes that extend beyond responding to acute stressors. Previous work has likened coping style variation to the FFM dimension of Openness, due to its reliance on the executive control of behavior—with more reactive individuals being more controlled and cautious (de Boer et al. 2017). In bonobos (*Pan paniscus*), Staes et al. (2017) found associations between Openness and approaches, as well as proximity, to a model leopard. Such predator responses parallel our measures of coping styles (Pritchard and Palombit 2022b, 2022a), indirectly linking coping style to Openness. Openness has also been likened to Exploration (Finkemeier et al. 2018; Gosling and John 1999), relevant as proactive individuals are typified by heightened exploratory behaviors in response to stress (Koolhaas et al. 1999; Verbeek et al. 1996). In humans, a ‘problem solving’ method of stress coping (Stanisławski 2019) has been linked to Activity (Gomà-i-Freixanet et al. 2021), from the alternative FFM (Zuckerman et al. 1991, 1993). Openness, in turn, has been associated with both the dimension of Activity (Singh and Kumar 2016) and higher physical activity (Sutin et al. 2016). Of relevance here, in humans, Openness has shown a negative association with allostatic load (Yoneda et al. 2023); just as coping style variation has had associations with allostatic load (Korte et al. 2005). Importantly, however, we note directional inconsistencies in how reactive and proactive individuals score on Openness (de Boer et al. 2017; Finkemeier et al. 2018; Gosling and John 1999). In sum, there is tentative evidence linking coping styles to variation in Openness, but we emphasize that coping style is unlikely to neatly map onto a single dimension (Finkemeier et al. 2018) and tendencies in aggression and perceived control (important to coping styles) are not represented at the level of domains in the FFM (Yoneda et al. 2023). Indeed, in humans, measures of coping style variation using a stressor covaried with aggression in men, but not in women (Gorka et al. 2016).

### Hypotheses and predictions

We collected data from field experiments, GC hormonal data, and behavioral observations obtained from wild olive baboons (*Papio anubis*) to study how individual differences in the SRS, interpreted via the coping style framework, are associated with fGCms. Using fGCm data, we can obtain profiles of expended unbound GCs, which in excess reflect energetic activation and may be associated partly with the SRS (Millspaugh and Washburn 2004; Sapolsky et al. 2000; Wingfield and Romero 2015). To the best of our knowledge, coping style and social support have never been explicitly examined together. This is surprising due to the aforementioned theoretical emphasis on the implication of individual differences with variation in the SRS, in addition to the securement and efficacy of social support (Cohen and Wills 1985; Mercado and Hibel 2017; Seyfarth et al. 2012; Swickert 2010) and social behavior more generally (Aplin et al. 2013; Koolhaas et al. 2017; Moyers et al. 2018; Snijders et al. 2014).

### Coping styles and GCs

Deriving mechanistic pathways of action for nuanced physiological systems in wild environments is challenging due to the complex action of glucocorticoids (Sapolsky 2000; Tkaczynski et al. 2019). We acknowledge that there are clear alternative hypotheses whereby, either, GCs are expected to drive coping style variation or coping style variation might be expected to alter GC expression. The former is supported given that GCs impact the cognitive function and processing of an organism (Pravosudov 2003; Sandi and Pinelo-Nava 2007; Sapolsky 1994) and operate in a capacity to influence ‘pending’ stressors (Sapolsky et al. 2000). The latter is supported given that other functional branches of the stress response can operate on a more rapid scale than GCs (Sapolsky et al. 2000). We acknowledge that it is difficult to test between these alternative hypotheses in a wild setting (Sapolsky et al. 2000; Tkaczynski et al. 2019). Thus, we broadly hypothesize that coping style variation will co-vary with GC concentrations. Despite this broad focus, we extend prior work (e.g., Tkaczynski et al. 2019; Ray and Sapolsky 1992; Sapolsky 1994; Sapolsky and Ray 1989) by disentangling measures of coping style variation from social behaviors.

A defining characteristic of the reactive coping style is behavioral flexibility, relative to the more patterned responses associated with the proactive coping style (Coppens et al. 2010; Koolhaas et al. 2010). Moderately elevated GC levels could facilitate flexibility through their capacity to improve the rapid intake and learning of novel information (i.e., enhancing memory (Pravosudov 2003) and facilitating synaptic plasticity (Sapolsky et al. 2000)). Through such an interpretation, GCs would be relevant upstream of coping style, influencing the cognitive processing underlying the distinct strategies. Thus, we predicted that individuals scoring on the more reactive end of the continuum will exhibit higher fGCms, relative to more proactive individuals (Prediction 1a [P1a]) (Bensky et al. 2017; Ferreira et al. 2016; Ibarra-Zatarain et al. 2016; Korte et al. 1992; Silva et al. 2010; Tkaczynski et al. 2019; Tudorache et al. 2013).

Chronically elevated GCs also enhance learning through conditioning, but suppress spatial learning (Sandi and Pinelo-Nava 2007). This is relevant given that proactive coping styles are characterized by consistently patterned responses to stimuli, analogous to learning via conditioning (Coppens et al. 2010; Koolhaas et al. 2010). While individuals with a reactive coping style spatially explore novel environments more slowly and thoroughly (Costantini et al. 2012; Verbeek et al. 1996). GCs could be an upstream process influencing coping styles with elevated values facilitating a patterned proactive response while suppressing the spatial learning indicative of reactive coping. Proactive coping styles are also characterized by low executive control and heightened aggressive tendencies—attempting to exert control over a stressor (Coppens et al. 2010; de Boer et al. 2017; Koolhaas et al. 2010). Male baboons who are unable to control aggressive tendencies have been shown to have high circulating plasma GCs (Sapolsky and Ray 1989). Thus, we predicted that individuals scoring on the more proactive end of the continuum will exhibit higher fGCms, relative to more reactive individuals (Prediction 1b [P1b]) (Costantini et al. 2012; Moyers et al. 2018; Sapolsky and Ray 1989).

### Social support and GCs

In line with prior work, we hypothesized that social support functions to buffer individuals from increased allostatic load (Beehner et al. 2005; Silk et al. 2009; Wittig et al. 2008). Primatological studies have generally focused on the benefits of social support in ameliorating the costs of activation of the SRS—stable, high-quality, relationships are associated with lower GCs (Beehner et al. 2005; Silk et al. 2009; Wittig et al. 2008). Although much focus has been directed towards female baboons, males also benefit from social bonds (Campos et al. 2020; Ray and Sapolsky 1992). Thus, we predicted that lower fGCms will be associated with focused quality relationships that function as social support—characterized by a high social investment among few social partners (i.e. low partner diversity scores)—versus weakened investments across many social partners (P2). We address this prediction using partner diversity, which is of utility here due to its prior use in foundational studies of social support in baboons (Crockford et al. 2008; Wittig et al. 2008) and its focus on allocation of socialization across partners rather than absolute investment (e.g., via raw rates or social network strength centralities) (Silk et al. 2013). Evidence for the benefits of social support are also likely to reflect reduced activation of the SRS due to non-supportive or aggressive interactions (Schrock et al. 2019; Seeman and McEwen 1996; Vandeleest et al. 2020).

### Social support and coping styles

Prior work has partially confounded social dynamics with the quantification of individual differences (Sapolsky 2000; Tkaczynski et al. 2019). Our measures of coping style do not include social metrics; thus, we can distinguish social effects relative to our measures of individual differences. Furthermore, the role of social support is important to revisit alongside coping style variation as the benefits of social support are theoretically expected to be influenced by individual differences (Sapolsky 1994; Seyfarth and Cheney 2013; Smith 2006; Kornienko et al. 2020; Mercado and Hibel 2017). Indeed, in nonprimates, coping style is associated with social network position, with reactive coping individuals having stronger bonds with fewer individuals, relative to proactive individuals (Aplin et al. 2013; Moyers et al. 2018; Snijders et al. 2014). Through this line of logic, reactive individuals would have stronger relationships, relative to proactive individuals that have diffuse relationships. This dynamic would result in lower fGCms in reactive coping individuals through the action of social support (P1b and P2). Thus, individual differences could be driving aspects of social support dynamics or be mediated wholly through the action of social support. These expectations, however, contradict evidence linking lower fGCms to more proactive coping styles (P1a) (Ferreira et al. 2016; Tkaczynski et al. 2019). Furthermore, in this population of baboons, coping style did not predict social position (dominance rank, centrality) within the group, but did influence patterns of association among strong proximate partners (Pritchard et al. 2023). Here, we seek to resolve these discrepancies by studying both dynamics in the same system to gain insight into whether coping style scores and measures of social support predict fGCms independently or interactively.

## Methods

Data were collected from November 2017 through April 2019, as part of the long-term ‘Project *Papio*’ (e.g., Danish and Palombit 2014; Lynch et al. 2017; Shur 2008) in Laikipia, Kenya (0°15′29″N 36°44′49″E). AJP collected data with the assistance of trained field staff on 44 adult baboons in two habituated groups: Kati-Kati (19 males; 9 females) and Shire (8 males; 8 females). We conducted field experiments to quantify individual coping style (P1a, P1b). We collected behavioral focal observations to obtain measures of social support (P2). Finally, we conducted non-invasive fecal sampling to estimate fecal glucocorticoid metabolite (fGCm) concentrations (P1a, P1b, P2).

Sampling intensiveness for focal data, experiments, and fecal sample collection is reported in the Supplementary Materials (Supplementary Tables 1 & 2). For each of the 44 individuals, we collected a mean of 130 focal follow samples. We conducted 62 experimental treatment trials on 32 individuals (25 males, 7 females). Finally, we collected 930 fecal samples from 43 subjects (Mean = 21.63 ± 3.70 *sd* of samples per subject) (P1a, P1b, P2). This number of samples is comparable to the median of 24 fecal samples per individual reported in published studies (Cavigelli and Caruso 2015).

AJP secured the necessary permits for animal observation, as well as sample collection and shipment from the: Kenyan Wildlife Service; National Commission for Science, Technology and Innovation; National Environment Management Authority; and Centers for Disease Control and Prevention; with support from the Institute of Primate Research and National Museums of Kenya. Approval was also obtained by Rutgers’ Institutional Animal Care and Use Committee (Protocol #16-039).

### Field experiments

Field experiments were conducted to quantify individual variation in coping style (P1a, P1b). Prior to starting the experiment, AJP verified four conditions were controlled for: (1) no conspecifics within 10 m of the subject; (2) no aggressive interactions involving the subject occurred in the preceding 10 min; (3) the subject was not participating in a consortship; and (4) the group did not experience any high arousal events in the preceding 20 min (for example, a large conflict event). To start the trial, a chicken egg was placed simultaneously with a model puff adder (*Bitis arietans*) in the anticipated travel path of a single baboon subject, specifically targeted when they were distant from conspecifics. The snake model was selected due to its demonstrated utility for measuring individual differences in fear-anxiety responses (Carter et al. 2012). Individually paired comparisons of these treatment experiments to control trials (an egg by itself; N = 30) revealed significantly higher measures of fear- and anxiety-associated behaviors in treatment trials (Pritchard and Palombit 2022a). Importantly, the inclusion of the egg presents multiple potential solutions, which is a necessary prerequisite for measuring coping style differences—i.e., variation in the response (Koolhaas et al. 1999, 2010; Pritchard and Palombit 2022a). Impulsive and confrontational responses are indicative of a proactive coping style, while strongly inhibited responses are indicative of a reactive coping style.

This experimental paradigm has been described elsewhere (Pritchard et al. 2023; Pritchard and Palombit 2022b, 2022a). Responses were video recorded in the field and the videos were later coded by an observer naive to the study’s purposes. We tested intraindividual consistency via the repeatability of responses (both Spearman’s ρ (Spearman 1904) and Kendall’s τ (Kendall 1938, 1945) > 0.20, with a mean of 0.45 and 0.39, respectively). Repeatable behaviors were reduced by means of a regularized exploratory factor analysis model (Jung and Lee 2011). The analysis revealed a single factor (accounting for 75% of the variance) that loaded on: latency to consume the egg after taking; duration of orienting towards the snake after taking the egg; duration of holding and consuming the egg (Pritchard and Palombit 2022a). We mean-aggregated coping style scores within each individual. Coping style scores were strongly associated with whether an individual decides to confront the stressor and take the egg in proximity to the stressor, as well as whether they ate the egg (Pritchard and Palombit 2022a). Factor scores covaried with latency to take the egg—a measure of impulsivity and a common indicator of coping style (Koolhaas et al. 1999; Pritchard and Palombit 2022a). This procedure resulted in individual coping style scores.

### Observational data

Individuals were randomly selected from a sequence list for 10-min focal follow sampling (Altmann 1974). During focal follows, observers recorded all aggressive, affiliative, and submissive behaviors (Ransom 1972; Strum 1982), along with the identity of all interaction partners. Behavioral data collection is additionally detailed elsewhere (Pritchard et al. 2023).

#### Shannon–Wiener diversity indices

During focals, we recorded actor-receiver specific grooming bouts to calculate Shannon–Wiener Diversity Indices (Wilson and Bossert 1971) (P2) using the R *vegan* packages *diversity()* function (v2.6-4) (Oksanen et al. 2024). These indices are a common measure of social support (Crockford et al. 2008; Wittig et al. 2008). This method provided a single Shannon–Wiener Diversity Index (SWDI) for each individual based on their grooming given. We relied on a single metric as we were interested in each individual’s capacity to maintain strong and stable bonds over time (i.e., social support), rather than intense but brief associations. The SWDI was derived to retain comparability with prior work. Importantly, this metric is derived from an information theory approach and accommodates uncertainty whereby individuals with more concentrated partner investment have greater certainty of partner investment relative to individuals with a greater diversity of grooming partners (Barnes and Spurr 1998; Kiernan 2014; Shannon and Weaver 1949). As reported in Silk et al. (2013), SWDI is calculated as:

$$H= {\sum_{i=1}^{R}}{{p}_{i}}{\log}{{p}_{i}}$$

where *p*_*i*_ is proportional grooming directed to each individual (*i*) which undergoes summation (Σ) across the set of social partners (*R*). Thus, SWDI is contingent on grooming duration *and* partner count. SWDI scores are more heavily influenced by partner count (i.e., richness—Kiernan 2014), rather than grooming duration (i.e., abundance—Kiernan 2014).

#### Ordinal dominance ranks

We recorded directional displacements (Bercovitch 1988) from focal follows and ad libitum observations (Altmann 1974). To calculate ordinal rank, we used the Percolation and Conductance method (Fujii et al. 2016; Pritchard et al. 2023; Vandeleest et al. 2016). This method allows for estimation of a hierarchy using a network approach that infers uncertainty based on transitive interactions and reversals. Our hierarchies exhibited intermediate-to-very steep hierarchies; greater detail in the rank structure of these groups is included elsewhere (Pritchard et al. 2023). This method provided a single ordinal rank measure for each individual, which we used in subsequent analyses.

### Fecal sample collection, extraction, and storage

#### Sample collection

Fecal samples were collected from July 29, 2018, to April 14, 2019, after arrival in the morning (7:00) and any time before noon. Afternoon fecal samples were not collected to avoid biases in fGCm attributable to circadian rhythms. Samples were collected ad libitum and were thus representative of general individual metabolite excretion. To avoid autocorrelations due to the gut-transit time for fecal samples, we implemented a two-day break for samples from the same subject. A small subset of samples (N = 16) were erroneously collected within that time interval. These samples were not excluded from processing or analysis.

#### Metabolite extraction and storage

We conducted solid phase extractions, which have been validated for long-term field storage (Beehner and Whitten 2004; Kalbitzer and Heistermann 2013; Shur 2008; Wasser et al. 2000). We utilized a protocol from the Lu Lab at Stony Brook University (Pers. communication, Lu 2018). After we conducted daily follows, we returned to our lodging to centrifuge samples; then we pipetted 2.0 ml of supernatant into a clean tube. We added 0.8 ml of 4:1 methanol:acetone and 5.6 ml of distilled water to the samples, then loaded them onto primed SepPak C18 cartridges. After packing these cartridges into individual airtight glass tubes with silica and sealing the tubes with parafilm, we shipped the samples to Erin Vogel’s Laboratory for Primate Dietary Ecology and Physiology, at Rutgers, the State University of New Jersey. After removal of the supernatant, we stored the remaining fecal matter with silica to desiccate the remaining contents. We transported the dried samples to the Institute of Primate Research, Nairobi, Kenya, for weighing to the nearest 0.0001 g.

### Radioimmunoassays

Radioimmunoassays were completed in the Vogel laboratory using a method validated for olive baboon fecal samples (Beehner and Whitten 2004; Kalbitzer and Heistermann 2013; Shur 2008). We used MP Biomedical Rat Corticosterone I^125^ kits (Catalog #07120103) following the included protocol. Samples were subjected to blowdown with compressed air before being reconstituted with the kit’s steroid diluent buffer.

#### Validation

We ran validations on the accuracy (spike and recovery) and specificity (parallelism) of the assays using our collected samples (Higham 2016). This was because prior hormone work from this population (Shur 2008) was conducted in a different lab. Validations were run using samples pooled by sex from extraction volumes of female and male samples throughout the study period. The accuracy showed a mean observed/expected concentration recovery of 84.46% ± 8.05sd (N = 6) for females, and 91.40% ± 12.57sd for males (N = 6). For the parallelism, the pooled samples within each sex were parallel with the standards. A sample volume of 40 μl, which was subjected to blowdown and reconstitution in 100 μl of buffer, was found to be close to 50% Binding/Total Binding for males (48.84% B/TB) and females (47.71% B/TB).

#### Inter- and intra-assay variation

We used MP Biomedical’s high and low quality controls to quantify inter-assay variation as a measure of precision (Higham 2016). Partway through laboratory analyses, MP Biomedical changed control lots and their associated concentrations. To accommodate the change in control lots, we report two sets of inter-assay coefficients of variation (CVs). The first set of runs (N = 16) had a low control CV of 8.72%, and a high control CV of 9.35%; the second set of runs (N = 12) were 4.91% and 4.82%, respectively. These values are below the 15% cut-off that we set, a priori. We reran samples that exceeded an intrasample CV of 15%; our retained samples had an average intra-assay CV of 3.65%.

### Climatological data

We collected data to control for environmental confounds in fGCm variation (Beehner and Bergman, 2017; Romero et al. 2009; Wingfield and Romero 2015). A relationship between fGCm concentrations and temperature has been demonstrated in several studies (Gesquiere et al. 2008; MacLarnon et al. 2015; Weingrill et al. 2004), though such an effect is not ubiquitous (reviewed in: MacLarnon et al. 2015). Extreme temperature introduces a thermoregulatory cost and can result in heightened fGCm concentrations (MacLarnon et al. 2015).

#### Temperature measurements

Field staff and AJP collected ambient maximum and minimum temperatures using a shaded external temperature probe from the east end of the Segera Ranchlands (0°10′21"N 36°53′38"E) between 17:00 and 21:00 local time. Due to errors during data collection, these climatic data were supplemented using data from the neighboring Mpala Research Centre (0°17′28"N 36°53′51"E) (Caylor et al. 2018), approximately eight miles from the Segera sampling locale. We ran a Welch’s t-test on a pooled sampling of max- and min-temperatures of overlapping data from eight days sampled at both sites. There were no significant differences in the overlapping subsample (t[29.87] = 0.35, p = 0.732; Cohen's d = 0.12, 95% CI [−0.57, 0.81]). For each fecal sample that we collected, we averaged the previous two days’ temperature readings to obtain maximum and minimum temperatures over an aggregate period relevant to a baboon’s gut transit time.

### Imputation of coping style scores

Only a subset of the samples (N = 699) were collected from animal subjects with coping style scores. To provide coping style estimates for subjects without scores we ran multiple imputation using the *mice* package (v3.16.0) (Buuren and Groothuis-Oudshoorn 2011). This approach facilitates running iterative versions of a single model with all samples, to address P1a, P1b, and P2. As opposed to running two separate models with one that includes a subset of the data to address P1a and P1b, and another with the full dataset (omitting coping style scores) to address P2.

To avoid informing coping style scores with social variables or fGCm concentrations, we limited predictive mean matching based on subjects’ sex and group. Predictive mean matching draws values from other subjects essentially based on a distance function (Little 1988). Visual inspection of the distribution of imputed coping scores showed similar distributions to the original data, as well as to data imputed using a simple sampling method. The similarity to randomly sampled data is to be expected given coping scores do not show an association with sex (Pritchard and Palombit 2022a) or group. We used a single initial imputation with 20 iterations for model comparison using *brm()* in the *brm* package. Once we had selected a final model structure, then we ran 100 imputations with 10 iterations for the final model using *brm_multiple()* in the *brms* package.

### Statistical analyses

For our analyses, we utilized Bayesian Regression Models using Stan (v2.20.4) (Bürkner 2017, 2018, 2021) through R (v4.3.1) (R Core Team 2021). All continuous variables were centered and rescaled by two standard deviations (Gelman 2008). We used lognormal distributions for all candidate models with the dependent variable of metabolite concentration of each sample in ng per g of dried fecal matter. We constructed models in a stepwise manner, first constructing a null model to confirm family fit, then including random effects (collection date, animal subject), before incrementally introducing temperature, then other confounding fixed effects (rank, group, sex), followed by the variables of interest (coping style scores and SWDI). As baboon social behavior is known to vary by sex (Strum 1987, 2012), we assessed interactions between SWDI, coping style, and sex. Within each of these five stages of model selection, we compared models using expected log pointwise predictive densities (ELPD in the *loo_compare()* function in *brms* [Bürkner 2017]) (Supplementary Table 3) and graphical posterior predictive checks (*pp_check()* function in the *bayesplot* package [v1.10.0] [Gabry et al. 2019]) (Supplementary Figs. 1 & 2). In the event that models performed similarly (≤ 2 se_diff from the elpd_diff estimates), we defaulted to simpler model structure unless one model had clear issues with fit. We retained both variables of interest for testing our predictions (SWDI and coping style scores), though top-performing models generally included these variables. During model selection, we used a warm-up of 200 on 2 chains, running for 1000 iterations.

The final model included fGCm concentrations as the response variable. Fixed effects included maximum temperature, minimum temperature, imputed coping style scores, SWDI, sex, and an interaction between SWDI and sex. Random effects included subject ID and collection day. We used weakly informative priors with a warm-up of 1000 on 4 chains, running for 3000 iterations and a thin of 2, across 100 imputations, resulting in 400,000 post-warmup draws. We visually assessed possible collinearity between fixed effects using pairs plots (Supplementary Fig. 2). Model convergence was assessed at Rhat = 1. Rhat (alternatively, $\widehat{R}$) values were generally 1, but the reported Rhats are often false positives in imputed models (Bürkner 2024). This is because chains across imputations may not align (Bürkner 2024). Thus, we confirmed that the submodels had Rhats = 1. We include summary statistics for all model parameters including estimates, estimate errors, and upper and lower credible intervals (CI). We include probability of direction values (pd) using the *p_direction* function in the *bayestestR* package (v0.13.2 Makowski et al. 2019) for clarity to researchers more familiar with frequentist analyses (Henzi et al. 2021), though we emphasize their lack of utility as a true cut-off (McElreath 2018). Model interactions were examined using the *emtrends()* function within the *emmeans* package (v 1.10.2 Lenth 2024). We generated whole model predictions to aid in interpretation using the *fitted()* function (Bürkner 2017). Posterior predictive plots were constructed from whole model predictions. Continuous variables not relevant to predictions were set to their means.

## Results

**Fig. 1:**
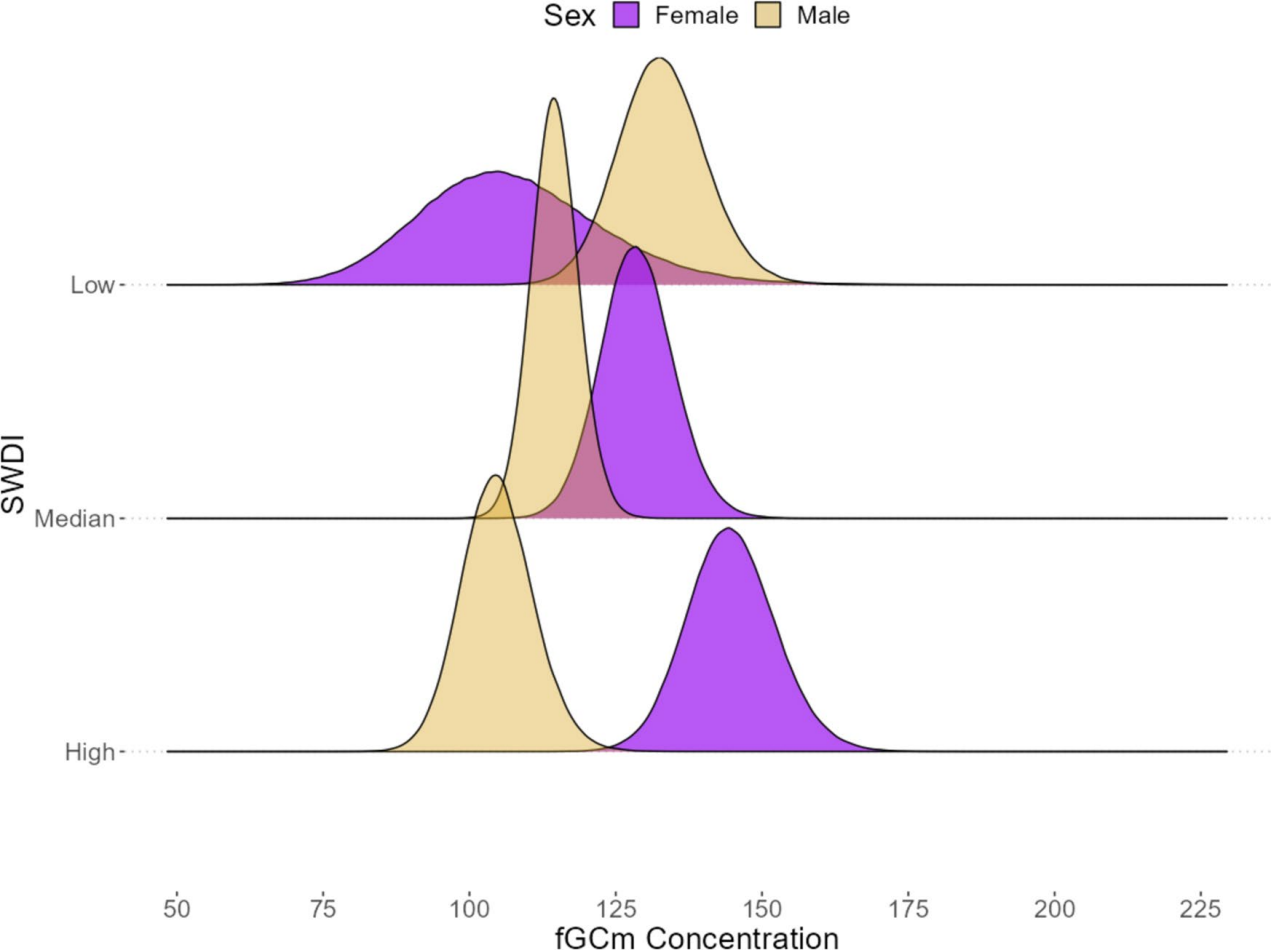
Posterior predictive plot of the interaction between grooming SWDI (y-axis) and sex (fill color and transparency) with fGCm concentration (x-axis). Density plots are the estimated probabilities generated from the full fit of the model posteriors at the minimum, mean, and maximum points of SWDI. The spread of the curve indicates the uncertainty of the predicted value. Note that the true data do not have females representing the minimum, thus the low SWDI density has higher uncertainty. We have included an additional interaction plot limiting the low SWDI to the minimum for females (Supplementary Fig. 3)

We used a dataset with imputed coping style scores in a *multiple_brm* model (N = 930 samples, mean per subject = 21.63 ± 3.70 *sd*) to test the influence of coping style and SWDI on fGCm concentrations. The full model explained 15.6% (Bayesian R^2^) of the variance in our dataset. Contrary to either P1a or P1b, coping style scores were not associated with fGCms (coping style score estimate = −0.05, 95% lower CI −0.14, upper CI 0.04; pd = 87.28%; Table 1). Neither more proactive nor more reactive coping style scores were associated with differences in fGCm concentrations. Social support was associated with fGCm concentrations (P2) (SWDI estimate = −0.14, 95% lower CI −0.25, upper CI −0.03; pd = 99.28%; Table 1). Importantly, the association between SWDI and fGCm concentrations was positive in males, and negative in females (interaction between SWDI and sex estimate = 0.32, 95% lower CI 0.09, upper CI 0.55; pd = 99.57%; Table 1; Fig. 1; Supplementary Fig. 3). Post hoc comparisons of the interaction showed identical outcomes for the reference group of males (SWDI trend = −0.14, lower HPD = −0.25, upper HPD = −0.03), while females were found to have a positive effect of SWDI, but were not credibly different from zero (SWDI trend = 0.18, lower HPD = −0.02, upper HPD = 0.38). Visual comparisons of posterior densities showed high estimate uncertainty among females with low SWDI.

**Table 1:** Final model output for fGCm concentrations, with Bayesian R^2^ estimates

	Estimate	Est. Error	l-95% CI	u-95% CI	Rhat	Bulk ESS	Tail ESS	Pd (%)
	Group-level effects							
Collection Day (149 levels)	0.17	0.03	0.13	0.22	1.00	217,288	300,344	–
Subject ID (43 levels)	0.07	0.03	0.01	0.13	1.00	106,691	140,610	–
	Population-level effects							
Intercept	4.62	0.03	4.56	4.68	1.00	338,666	362,231	100.00
Temperature Maximum	0.11	0.04	0.02	0.19	1.00	328,413	363,539	99.12
Minimum	−0.07	0.04	−0.16	0.02	1.00	330,386	362,058	94.62
Sex (F)	0.10	0.06	−0.02	0.21	1.01	58,554	354,124	95.48
SWDI	−0.14	0.06	−0.25	−0.03	1.00	89,205	351,463	99.28
Interaction (SWDI:Sex[F])	0.32	0.12	0.09	0.55	1.01	55,554	350,086	99.57
Coping Scores	−0.05	0.05	−0.14	0.04	1.14	1820	6827	87.28
	Family specific parameters							
Sigma	0.46	0.01	0.44	0.49	1.00	280,057	333,464	–
	Bayesian R-squared							
Conditional	0.156	0.026	0.106	0.207	–	–	–	–
Marginal	0.049	0.015	0.023	0.082	–	–	–	–

Column header abbreviations are as follows: *CI* Credible Intervals, *ESS* Effective Sample Size, *Pd* Probability of Direction. ESS and Rhat provide estimates for goodness-of-fit; CI and Pd provide estimates of meaningful differences

Female and male baboons did not differ in their fGCm concentrations, though females had slightly elevated fGCm concentrations relative to males (Table 1). During our study, maximum daily temperature ranged from 20.10 to 39.40 °C (29.94 °C M ± 2.49*sd*) and minimum daily temperature ranged from 6.10 to 26.20 °C (12.70 °C M ± 3.24*sd*). Maximum and minimum temperature were not correlated with one another (*r* = −0.05). Maximum daily temperature was associated with fGCm concentrations, such that higher maximum daily temperatures resulted in higher fGCms, relative to cooler maximum daily temperatures (Table 1). Minimum daily temperature was not associated with fGCm concentrations (Table 1).

Because coping style was unassociated with fGCms and to confirm that the imputations did not overly influence our findings, we ran a similar model on the original dataset omitting coping style (Supplementary Figs. 4 & 5; Supplementary Table 4). Coefficients for the remaining variables were similar to those found in the imputed model (≤ ± 0.02). Credible associations were consistent between the two models (i.e., SWDI, its interaction with sex, and maximum temperature) (Supplementary Table 4).

As SWDI can be influenced by both the number of partners and their relative investment, we ran a post hoc analysis of similar structure using grooming out-degree and out-strength. Grooming network degree was defined as the number of unique partners each individual groomed, while strength was the number of these partners, inclusive of the duration of grooming. SWDI was more highly correlated with out-degree (*r* = 0.90 in males, and 0.92 in females) than out-strength (*r* = 0.64 in males, and 0.37 in females). Males and females did not markedly differ in mean out-strength per edge (i.e., strength divided by degree; males = 3.75 M ± 3.06*sd*; females = 5.13 M ± 3.07*sd*). Because strength can covary with the number of partners, we included an interaction between strength and degree, degree and sex, as well as strength and sex; we did not include the three way interaction term. Model fit was acceptable, as previously described for models built with a priori expectations (Supplementary Table 5; Supplementary Figs. 6 & 7). Our full model with SWDI was similar in fit based on the *se* and ELPD differences assessed using *loo_compare()* (elpd_diff = −1.5, se_diff = 1.8, reference = SWDI model without imputed data). Although strength and degree estimates were linearly correlated (*r* = 0.77), inspection of posteriors using *pairs()* and examination of variance inflation factors did not indicate strong multicollinearity (Supplementary Fig. 7; Supplementary Table 6).

Our post hoc model indicated that differences in fGCm concentrations were attributable to grooming out-degree with more certainty than our SWDI estimates (degree estimate = −0.30, 95% lower CI −0.52, upper CI −0.08; pd = 99.55%). We also found an SWDI by sex interaction with the direction of its association showing the same dynamic as for our SWDI model (interaction between degree and sex estimate = 0.38, 95% lower CI 0.07, upper CI 0.69; pd = 99.27%): males with low degree had relatively higher fGCm concentrations compared to males with high degree, while females showed the opposite association, though it was not credibly different from zero (out-degree trend = 0.08, lower HPD = −0.12, upper HPD = 0.28). We did not find the same associations for grooming out-strength (estimate = 0.19, 95% lower CI −0.02, upper CI 0.40; pd = 96.59%), nor evidence of a strength:sex interaction (estimate = −0.16, 95% lower CI −0.42, upper CI 0.11; pd = 88.63%). We present full model results in the Supplementary Materials (Supplementary Table 6).

## Discussion

We found that fGCm concentrations were not associated with coping style scores (P1a, P1b), in contrast to prior research suggesting this covariance in other taxa (Bensky et al. 2017; Costantini et al. 2012; Ferreira et al. 2016; Ibarra-Zatarain et al. 2016; Korte et al. 1992; Moyers et al. 2018; Silva et al. 2010; Tkaczynski et al. 2019; Tudorache et al. 2013). Even so, our results agree with numerous studies that report a null result in this association (Baugh et al. 2017a, b; Ferrari et al. 2020; Kanitz et al. 2019; Qu et al. 2018; Vindas et al. 2017; Westrick et al. 2019; Wong et al. 2019). We found an effect of social support on fGCm concentrations in olive baboons (P2), in agreement with prior work on the closely related chacma baboons (Crockford et al. 2008; Wittig et al. 2008). Importantly, however, we provide evidence that males and females have an opposite association between a measure of social support (SWDI) and fGCms—an association which is likely driven by the number of partners subjects groomed. Here we discuss the implications of no association between coping style scores and fGCms, and examine what might be driving an interaction between SWDI and sex with regards to fGCms.

### Coping style and glucocorticoids

The lack of an association between fGCms and coping style scores (P1a, P1b) contrasts with several other mammalian studies (Costantini et al. 2012; Ferreira et al. 2016; Tkaczynski et al. 2019). These findings emphasize that: (a) coping style scores are independent of HPA activity (Qu et al. 2018; Santicchia et al. 2019); (b) differences in coping style might act on different components of the HPA axis that are not detectable via fGCms, such as mineralocorticoid neural receptor expression (Baugh et al. 2017a, b); or, (c) HPA activity is a consequence of different behavioral responses to challenges (Costantini et al. 2012; Koolhaas et al. 2010). To clarify the last point, individuals that are entirely risk averse are predicted to have a reactive coping style and, thus, avoid interacting with a stressor entirely. This behavioral strategy is likely to limit GC production, but only in contexts where the individual can avoid challenges. This rationale might explain why different studies have reported such a wide variety of findings regarding the association between coping style and GC concentrations. That is, HPA activity covaries both with individual tendencies, and with the nature of the stressor and its circumstances. For example, individuals experiencing an immobile snake can immediately withdraw if they choose to do so, but agonistic socio-sexual encounters with mobile and motivated conspecifics may be difficult to avoid.

GCs are influenced by many extrinsic and intrinsic variables (Wingfield and Romero 2015). Furthermore, GCs can be both the cause and consequence of physiological effects (Sapolsky et al. 2000). As such, GC action could in principle be too multifactorial; contingent on numerous, biological, physiological, as well as environmental interactions to be markedly influenced by individual coping styles in wild primate species. Such an assertion, however, must accommodate known or theoretical associations between personality differences, sensu lato, and allostatic load (e.g., Korte et al. 2005; Yoneda et al. 2023). In humans, a fundamental aspect of allostatic load is *perceived* stress (Yoneda et al. 2023). Comparable investigations of nonhumans would require assessments that more directly measure perceptions and future expectations. For example, Sapolsky measured males’ capacity to differentiate the “tone” of interactions (neutral vs. aggressive) and their future outcomes (probability of win vs. loss) (Sapolsky 1994). Furthermore, if we assume that coping styles emerge as a consequence of frequency dependent trade-offs (e.g., Carere et al. 2010; Wolf and Weissing 2010) or are contextually or situationally adaptive (e.g., Chittka et al. 2009; Koolhaas et al. 2017; Korte et al. 2005), then we might expect inconsistent or nuanced advantages for each end of the coping style continuum. Isolating the ecological contexts under which the extreme ends of the coping style continuum outperform each other would provide insight into the ecological reality of such theory. Only then can we examine whether nuanced differences in alternative solutions (i.e., coping styles) are similarly advantageous across longer time periods that span varied contexts.

### Social support covaries with fGCms, but interacts with sex

We found an association between social support (SWDI) and fGCm concentrations (P2). Importantly, however, the nature of this relationship is contingent on the sex of the animal: males had a negative association between SWDI and fGCm, while females had a positive, but not credibly meaningful, association. Post hoc models indicate that the dynamics driving this association are attributable to out-degree, rather than strength. That is, it is the number of associations individuals invest in that is driving these associations. Based on known qualities of SWDI as a metric (Barnes and Spurr 1998; Kiernan 2014), the influence of degree (i.e., richness) is unsurprising—but, on the other hand, has not been extensively emphasized in relevant papers on social support in baboons. As such, here we discuss the implications and mechanisms that might underlie these associations.

Our results partially substantiate early work by Sapolsky and Ray. They found that, in olive baboons at Maasai Mara, Kenya, high ranking males with higher rates of grooming with consorting and non-consorting females had lower plasma GCs, relative to males with lower rates of grooming (Ray and Sapolsky 1992; Sapolsky and Ray 1989). In the current study, the majority of male grooming interactions were within mixed sex dyads and subsumed sexual consortships and heterosexual friendships. As grooming interactions often partly characterize friendships (Lemasson et al. 2007; Smuts 2017), it may be tempting to invoke these unique dyadic pairings as a partial source of grooming and, consequently, as a contributor to our associations between SWDI and fGCms. In this same population of baboons, however, Shur (2008) reported a rise in male fGCms in the 8 weeks following the birth of an infant to a female and her initiation of friendships. Thus, one interpretation might suggest that, for males, a greater multitude of friendships during this critical period of infant development is unlikely to be the most pronounced source of our findings—though higher resolution and more long-term behavioral data would be necessary to examine this dynamic. In this population, rank was not correlated with male SWDI (*r* = 0.03), indicating that high ranking males, who are more likely to secure consorts and have a greater number of friends, were not necessarily exhibiting higher SWDI. Indeed, rank did not contribute to model fit throughout model selection.

While we have focused on the effect of social support for males, we acknowledge that an energetic hypothesis could also explain this relationship. Alberts et al. (1996) observed that male yellow baboons participating in sexual consortships had shorter daily travelling and briefer feeding bouts, relative to non-consorting males. This observation is relevant because consorting males are more likely to be engaged in grooming bouts, relative to non-consorting males (Rasmussen 1983). Though this interpretation ignores the increased energetic exertion consorting males may be expending in contest competitions (Gesquiere et al. 2011). Future work should focus on male activity with respect to grooming, consorting, foraging, and feeding to parse the causative relationship of our findings, especially in reference to female behavior.

In our study groups, we also found an interaction indicative of sex differences in the association between fGCms and SWDI, likely driven by differences in grooming out-degree. Despite an overall negative association between SWDI and fGCms with males as our reference group, our interaction reveals that females exhibited a positive association between the two variables; though high uncertainty among females with low SWDI reduced credibility of this finding. Even so, this finding was directionally consistent with prior work where females with lower SWDI had reduced fGCms, relative to females with higher SWDI (Crockford et al. 2008; Wittig et al. 2008). We recognize, however, that social support is a dynamic process; the strength of the association between fGCm and SWDI might vary across reproductive states (Crockford et al. 2008) and instability in the male hierarchy (Wittig et al. 2008).

SWDI was selected due to its precedence in studies of support among baboons, however, our work emphasizes that other metrics of social support might provide more intuitive and interpretable metrics. Because social behavior is quite sex-differentiated in olive baboons (Strum 1987, 2012), parsing how the mechanisms that underlie our associations are acting distinctly between the sexes is challenging. Furthermore, as there were few females with very low SWDI or degree, and few males with very high degree, it is unclear if these sex differences emerge through a non-linear association between a social variable and fGCms. To clarify, extremely few and many social partners could both result in heightened fGCms relative to an intermediate number of social partners, resulting in a U-shaped relationship with fGCms. Extremely high-resolution behavioral data paired with fine-grained environmental and energetic sampling over a long-term study period are likely prerequisites for resolving these dynamics, including the sex differences described here.

### Limitations

We acknowledge a limitation in the temporal association between the numerous fecal samples collected and the single measures for rank, coping style, SWDI, out-strength, and out-degree. Estimates of rank steepness and repeatability, using the randomized Elo-rating package (Sánchez-Tójar et al. 2018), indicated that our hierarchies were intermediate-to-very steep with intermediate-to-high repeatability (Pritchard et al. 2023). Quantitative analyses of data indicate that these dynamics are likely a product of social uncertainty, rather than insufficient data (Pritchard et al. 2023). Thus, our rank estimates may be more dynamic than a single ordinal rank metric can represent. Indeed, five of our subjects died during our study (Supplementary), which can alter rank even as a passive process. Partitioning the data, however, would reduce the data below recommended interaction ratios for calculating rank (Pritchard et al. 2023; Sánchez-Tójar et al. 2018). Similarly, experimental events for measuring coping style and the collection of social data could be increased in sampling density. The former change might alter contextual noise present in individual trials, but drastically increases the logistic burden of similar work.

## Conclusion

Consistent individual differences in response to a stressor (coping style) did not covary with fGCm concentrations in these baboons. We acknowledge, however, that understanding individual differences in fGCms is challenging, especially given various unknowns such as variation in the action or abundance of GC receptors (Wingfield and Romero 2015). Thus, we emphasize the importance of continuing to examine individual differences in GCs. Importantly, we documented an effect of social support, but with an interaction between subject’s sex and SWDI. Males that had higher grooming diversity had lower glucocorticoid metabolite concentrations relative to males with a lower grooming diversity; females exhibited the opposite dynamic, but not with high certainty. In males, it remains an outstanding question as to whether this is observed association due to a socially-induced reduction of GC levels. Even so, we emphasize the importance of these data for elucidating the contrasting influence that social support can have and extend caution into the assumption that social support is a ubiquitously buffering process. Rather, the interplay of balancing investment across the appropriate number of partners could have important specificity with regards to the study subject’s sex.

## Supplementary Information

Below is the link to the electronic supplementary material.

Supplementary file 1 (PDF 2155 KB)

## Data Availability

The data that support the findings of this study are openly available in DataDryad at https://doi.org/10.5061/dryad.q2bvq83r9.
