# Supplementary material for: The relationship of coping style and social support variation to glucocorticoid metabolites in wild olive baboons (Papio anubis)
Source: Primates. 2024 Dec 12;66(1):87–102. doi: 10.1007/s10329-024-01172-2 (PMC11735542; doi:10.1007/s10329-024-01172-2)
Supplement: Supplementary file 1 — (PDF 2155 KB) [file 10329_2024_1172_MOESM1_ESM.pdf]

Supplementary Materials

The Relationship of Coping Style and Social Support Variation to Glucocorticoid Metabolites in Wild Olive Baboons (*Papio anubis*)

Alexander J. Pritchard[a,b,c] *, Erin R. Vogel[a,b], Rosemary A. Blersch[c], Ryne A. Palombit[a,b]

*Corresponding author: ajpritchard@ucdavis.edu ; ORCiD 0000-0003-4922-3347

[a] Center for Human Evolutionary Sciences, Rutgers, The State University of New Jersey, New Brunswick, NJ, U.S.A.

[b] Program in Human Evolutionary Sciences, Department of Anthropology, Rutgers, The State University of New Jersey, New Brunswick, NJ, U.S.A.

[c] California National Primate Research Center, University of California, Davis, Davis, CA, U.S.A.

Supplementary Table 1. Kati-Kati group membership and sampling intensiveness, per ID. Social rank is ordinal, within group and sex. Asterisked individuals disappeared during the data collection period (e.g., due to deaths or emigrations).

ID Code	Sex	Social Rank	Fecal Samples	Focal Hours	Experimental Treatment Counts
CB	F	8	22	23.03	2
EV	F	7	22	23.63	0
GW	F	3	22	23.69	1
ID	F	9	22	22.77	0
LH	F	6	22	23.25	0
LL	F	4	23	23.02	2
NU	F	1	23	23.08	2
NV	F	5	22	23.15	0
QN*	F	2	12	16.74	0
BD*	M	5	16	18.67	0
CT	M	15	22	22.88	2
DS	M	11	23	23.65	2
ED*	M	13	16	19.86	3
JR	M	4	22	23	2
LB	M	6	23	23.17	2
MA	M	19	23	22.9	3
OV	M	16	23	23.23	1
PQ	M	8	24	23.17	2
QO	M	14	23	23.13	3
RC	M	18	24	23.06	2
VA	M	10	22	23.17	3
WB	M	3	23	23.2	3
WR	M	12	22	23.17	2
WS	M	17	23	23.57	2
XB	M	1	23	23.24	2
XD	M	7	24	23.32	2
XU	M	2	23	23	1
ZK	M	9	25	23.38	1
**Median**:			23	23.16	2

Supplementary Table 2. Shire group membership and sampling intensiveness, per ID. Social rank is ordinal, within group and sex. Asterisked individuals disappeared during the data collection period (e.g., due to deaths or emigrations).

ID Code	Sex	Social Rank	Fecal Samples	Focal Hours	Experimental Treatment Counts
DV	F	3	22	23.41	2
IL	F	5	21	23.99	0
KS*	F	4	03	12.33	0
MW	F	1	24	23.17	0
OH	F	8	21	23.57	2
VD	F	6	23	23.43	1
XT	F	7	23	23.90	0
ZB	F	2	21	23.07	0
BY	M	2	22	23.71	2
BZ	M	3	23	23.58	1
DC*	M	4	00	10.17	2
GV	M	6	24	23.33	2
OO	M	5	23	23.17	2
QA	M	8	23	23.69	0
QR	M	7	22	23.34	2
SQ	M	1	21	23.25	1
**Median**:			22	23.375	1

Supplementary Table 3. Fitted model comparisons for candidate models with imputed coping style scores based on ELPD, generated using *loo_compare()*. The best fit model has the largest ELPD and is presented in the first row; the selected model is italicized.

Model Identifier*	Stage**	Differences		Fixed Effects			Random Effects
		ELPD	se	Predictions	Other	Interactions	
18	*5*	*0*	*0*	*SWDI, Coping*	*Max & Min Temp., Sex*	*Sex * SWDI*	*Collection Day, ID*
13	4	-1	2.7	–	Max & Min Temp., Sex	–	Collection Day, ID
20	5	-1	1.6	SWDI, Coping	Max & Min Temp., Sex	Sex * Coping * SWDI	Collection Day, ID
21	5	-1.3	1.7	SWDI, Coping	Max & Min Temp., Sex	Sex * SWDI	Collection Day, ID, Run Date
9	3	-1.8	3.6	–	Max & Min Temp.	–	Collection Day, ID
16	5	-1.9	2.2	SWDI, Coping	Max & Min Temp., Sex	–	Collection Day, ID
17	5	-2	2.4	SWDI, Coping	Max & Min Temp., Sex	SWDI * Coping	Collection Day, ID
19	5	-2.2	2.3	SWDI, Coping	Max & Min Temp., Sex	Sex * Coping	Collection Day, ID
15	4	-2.3	2.8	–	Max & Min Temp., Sex, Rank	Sex * Rank	Collection Day, ID
12	3	-2.4	3.7	–	Max & Min Temp.	–	Collection Day, ID
10	3	-2.6	3.6	–	Max Temp.	–	Collection Day, ID
11	3	-2.7	4	–	Min Temp.	–	Collection Day, ID
14	4	-3.2	2.7	–	Max & Min Temp., Sex, Rank	–	Collection Day, ID
5	2	-4.1	4.2	–	–	–	Collection Day, ID
7	2	-4.2	4.2	–	Group	–	Collection Day, ID
3	2	-13.7	8.8	–	Collection Day (spline)	–	ID
8	2	-19.7	9.4	–	Collection Day (linear), Group	Collection Day (linear) * Group	ID
4	2	-25.1	8.9	–	Collection Day (linear)	–	ID
6	2	-25.4	8.9	–	Collection Day (linear), Group	–	ID
2	2	-33.9	9.1	–	–	–	ID
1	1	-43.5	10.6	–	–	–	–

* In sequential order of writing

** Top-performing model in each stage was used as the base model in subsequent stages. In order, these are models: 5, 9, 13, and 18.

Supplementary Figure 1. Nine randomly selected graphical posterior predictive checks to assess model fit for the full model, across sampled imputations.

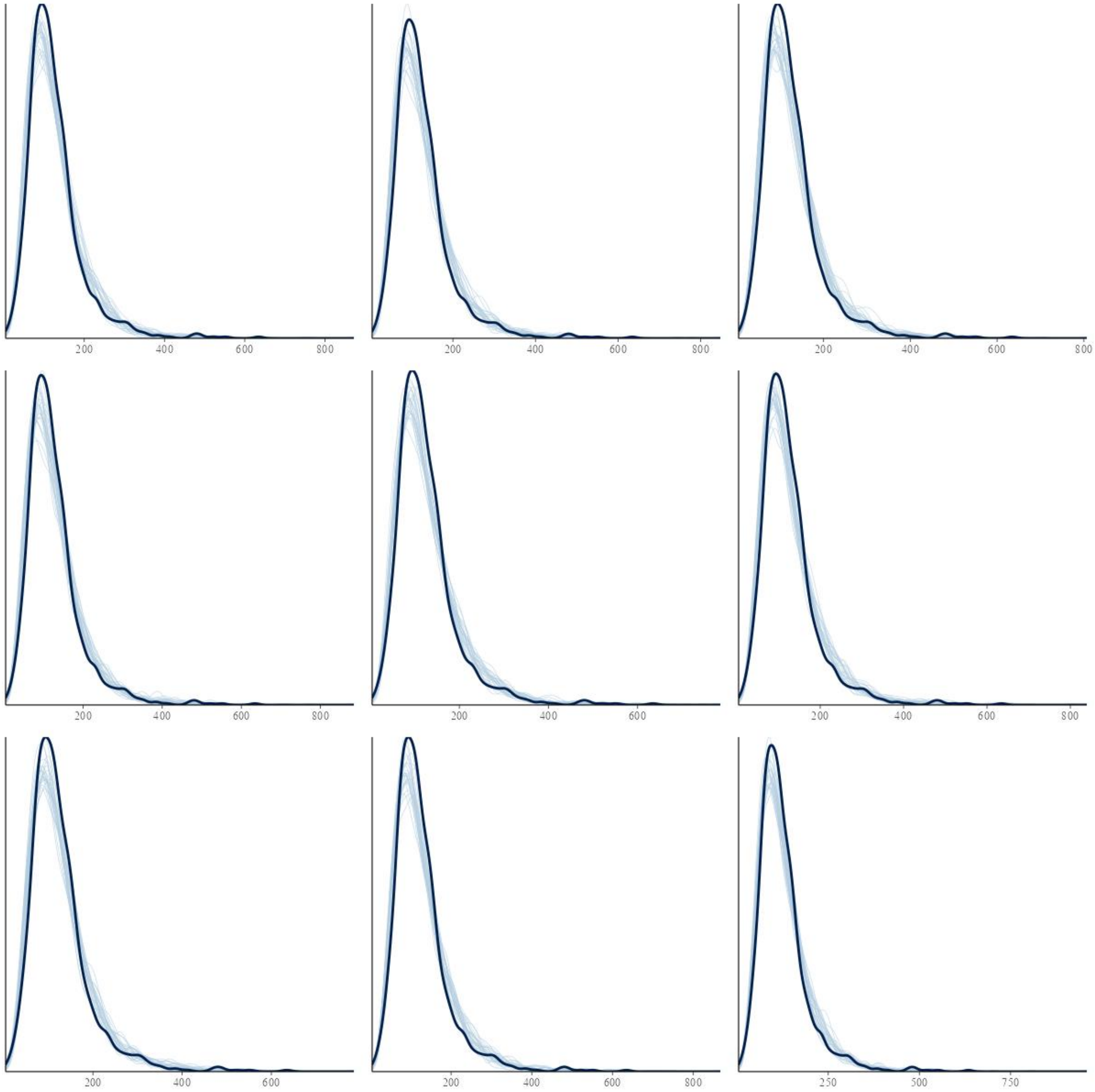

Supplementary Figure 2. Four randomly selected pairs plots to assess possible multicollinearity for the full model, across imputations.

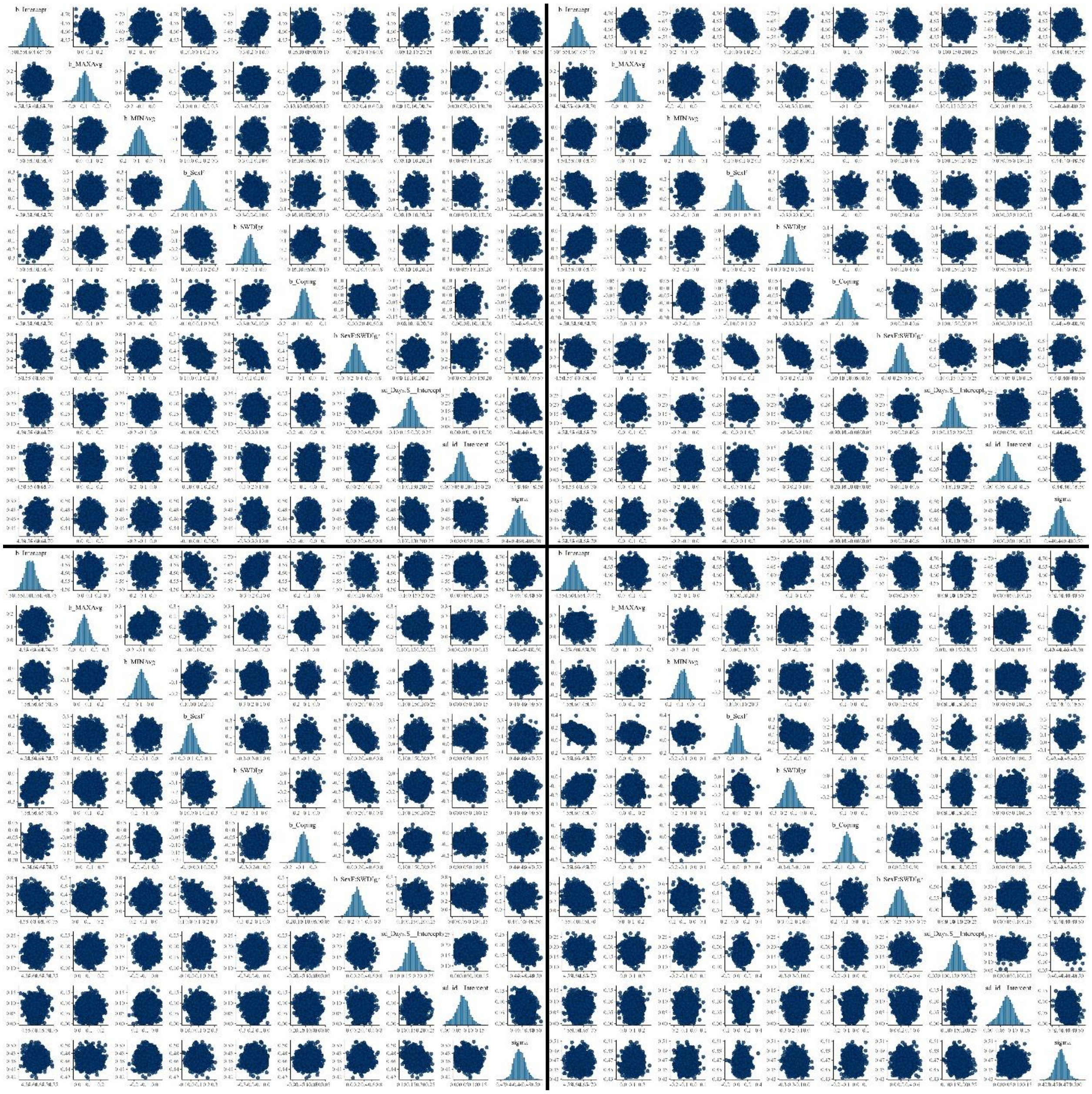

Supplementary Figure 3. Density plot of the interaction between grooming SWDI (y-axis) and sex (fill color and transparency) with fGCm concentration (x-axis). Note that this plot is identical to Figure 1 in the manuscript, but with low SWDI estimated from the point of females' minimum.

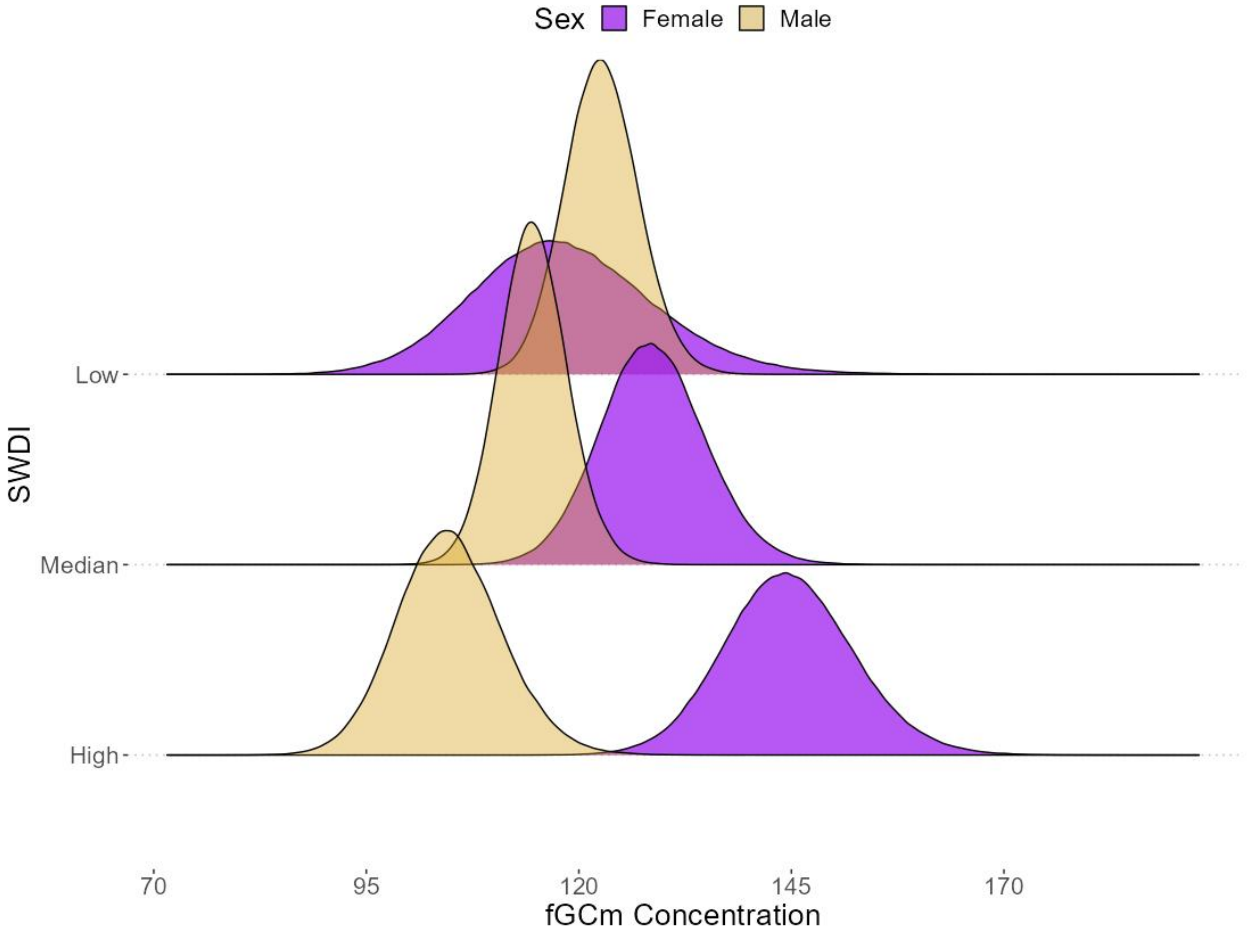

Supplementary Figure 4. Graphical posterior predictive checks to assess model fit for the full model with the entire dataset, excluding coping style scores.

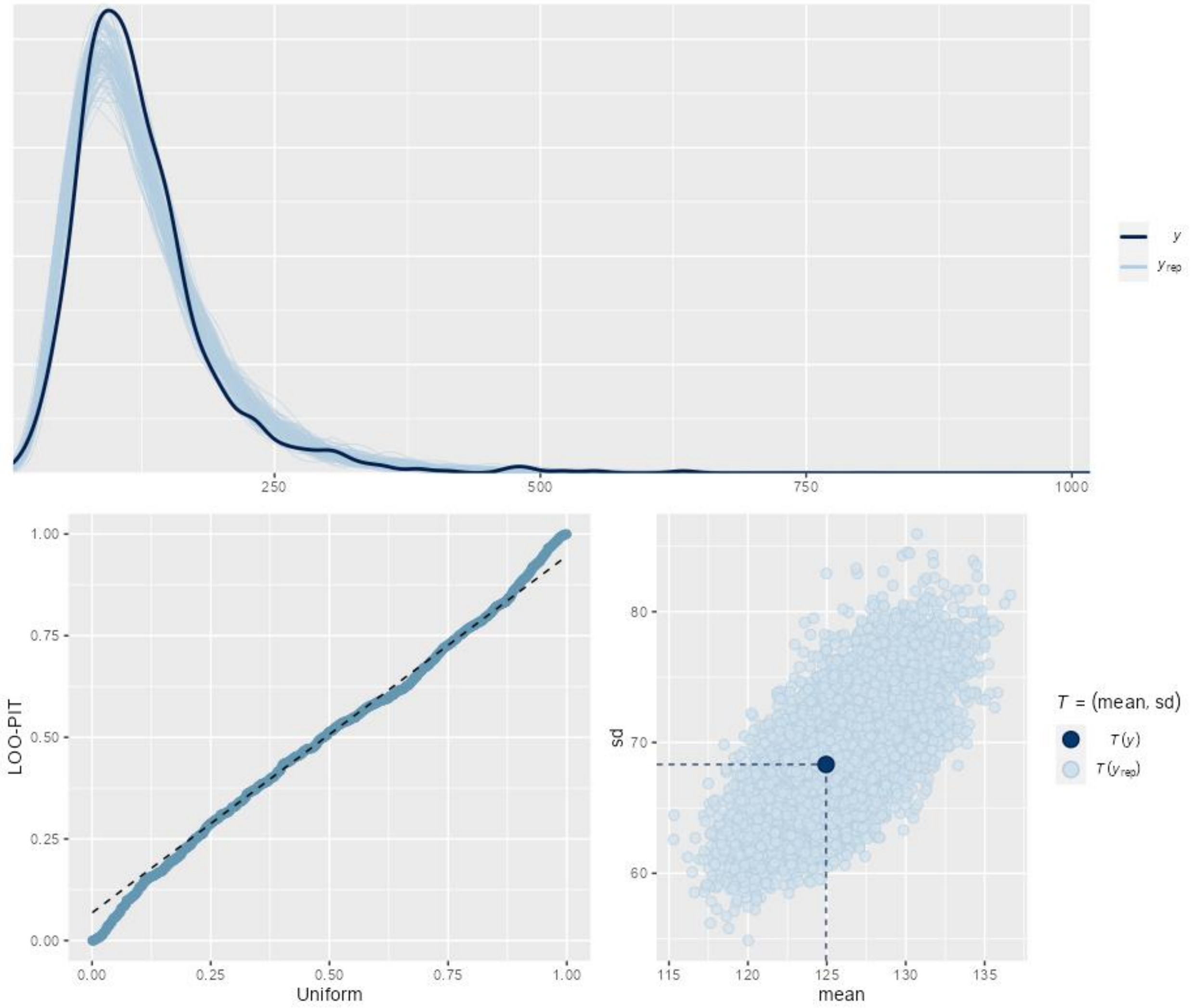

Supplementary Figure 5. Pairs plot to assess possible multicollinearity for the full model with the entire dataset, excluding coping style scores.

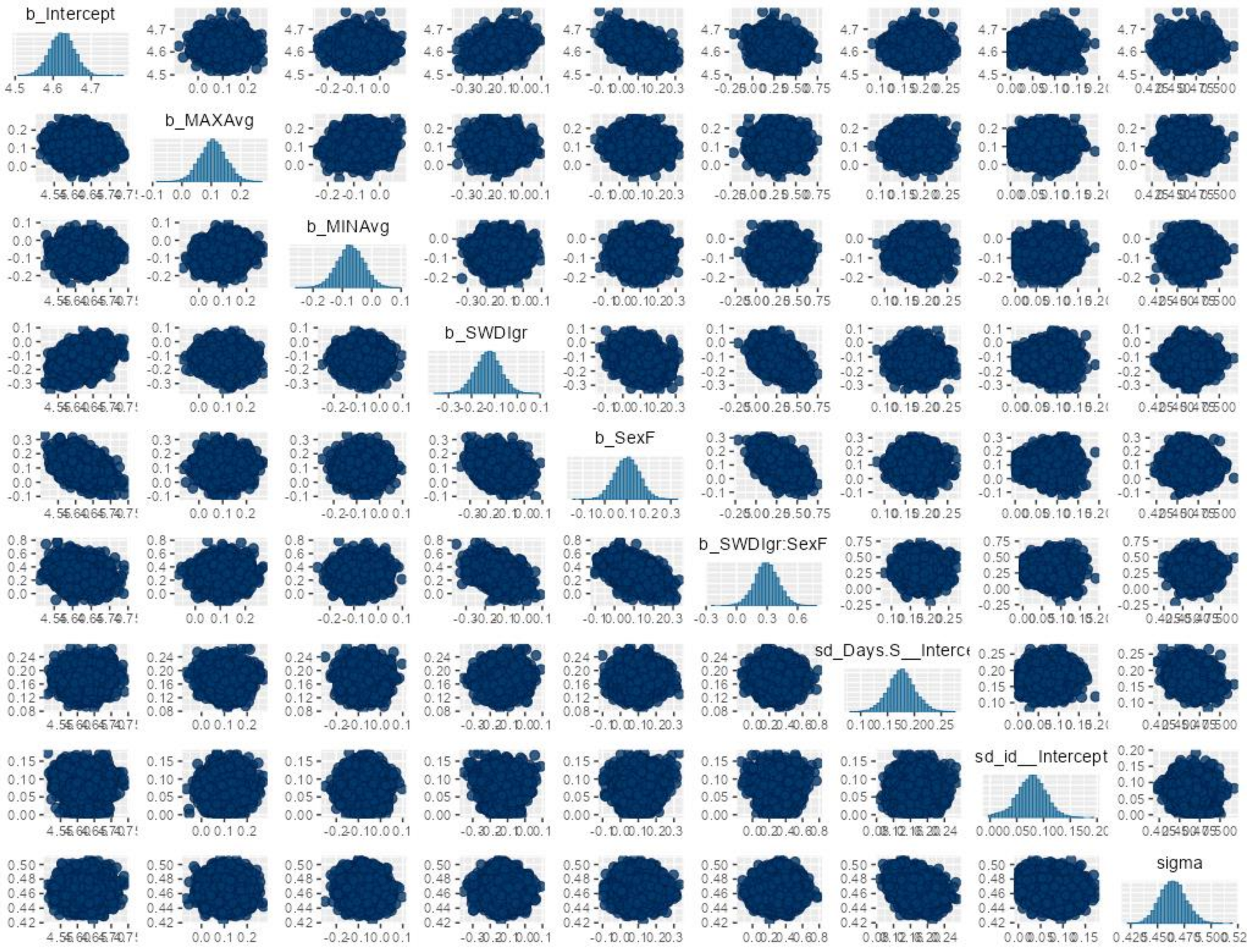

Supplementary Table 4. Model output from the final Bayesian mixed-effects model, excluding coping style as a predictor and without data imputations. Run with 4 chains, each with 6000 iterations, a warmup of 1000, thin of 2, resulting in 10,000 post-warmup draws.

	Estimate	Est. Error	l-95% CI	u-95% CI	Rhat	Bulk ESS	Tail ESS	pd
	**Group-Level Effects**							
Collection Day (149 levels)	0.18	0.03	0.13	0.23	1	5462	7122	–
Subject ID (43 levels)	0.08	0.03	0.02	0.13	1	4165	4414	–
	**Population-Level Effects**							
Intercept	4.62	0.03	4.56	4.69	1	8416	8423	100.00
Temperature Maximum	0.11	0.05	0.02	0.20	1	7419	7781	99.07
Minimum	-0.07	0.04	-0.16	0.02	1	7152	8629	94.51
Sex (F)	0.10	0.06	-0.01	0.21	1	7834	8615	96.38
SWDI	-0.13	0.06	-0.24	-0.02	1	8342	8781	98.86
Interaction (SWDI:Sex[F])	0.30	0.12	0.07	0.52	1	7149	8225	99.44
	**Family Specific Parameters**							
Sigma	0.46	0.01	0.44	0.49	1	7152	8123	–
	**Bayesian R-squared**							
Conditional	0.156	0.025	0.107	0.206	–	–	–	–
Marginal	0.046	0.015	0.020	0.079	–	–	–	–

Model Notes: There were no divergent transitions after warmup.

Supplementary Table 5. Model output from a Bayesian mixed-effects model that included grooming out-degree and out-strength as predictors. Run with 4 chains, each with 6000 iterations, a warmup of 1000, thin of 2, resulting in 10,000 post-warmup draws.

	Estimate	Est. Error	l-95% CI	u-95% CI	Rhat	Bulk ESS	Tail ESS	pd
	**Group-Level Effects**							
Collection Day (149 levels)	0.18	0.03	0.13	0.23	1	5563	7861	–
Subject ID (43 levels)	0.08	0.03	0.02	0.14	1	3897	3786	–
	**Population-Level Effects**							
Intercept	4.62	0.04	4.55	4.70	1	9590	8996	100.00
Temperature Maximum	0.10	0.05	0.02	0.19	1	9154	9369	99.07
Minimum	-0.07	0.05	-0.16	0.02	1	9386	8703	94.66
Out.Deg	-0.30	0.11	-0.52	-0.08	1	8820	8944	99.55
Out.Str	0.19	0.11	-0.02	0.40	1	8707	8647	96.59
Sex (F)	0.12	0.06	0.01	0.23	1	9311	9257	98.52
Interaction (Out.Deg:Out.Str)	0.00	0.12	-0.24	0.23	1	9420	8753	50.45
(Out.Str:Sex[F])	-0.16	0.13	-0.42	0.11	1	8339	8595	88.63
(Out.Deg:Sex[F])	0.38	0.16	0.07	0.69	1	8467	8583	99.27
	**Family Specific Parameters**							
Sigma	0.46	0.01	0.44	0.49	1	6225	7390	–
	**Bayesian R-squared**							
Conditional	0.159	0.025	0.111	0.210	–	–	–	–
Marginal	0.053	0.016	0.025	0.088	–	–	–	–

Model Notes: There were no divergent transitions after warmup.

Supplementary Figure 6. Graphical posterior predictive checks to assess model fit for the out-degree and out-strength model.

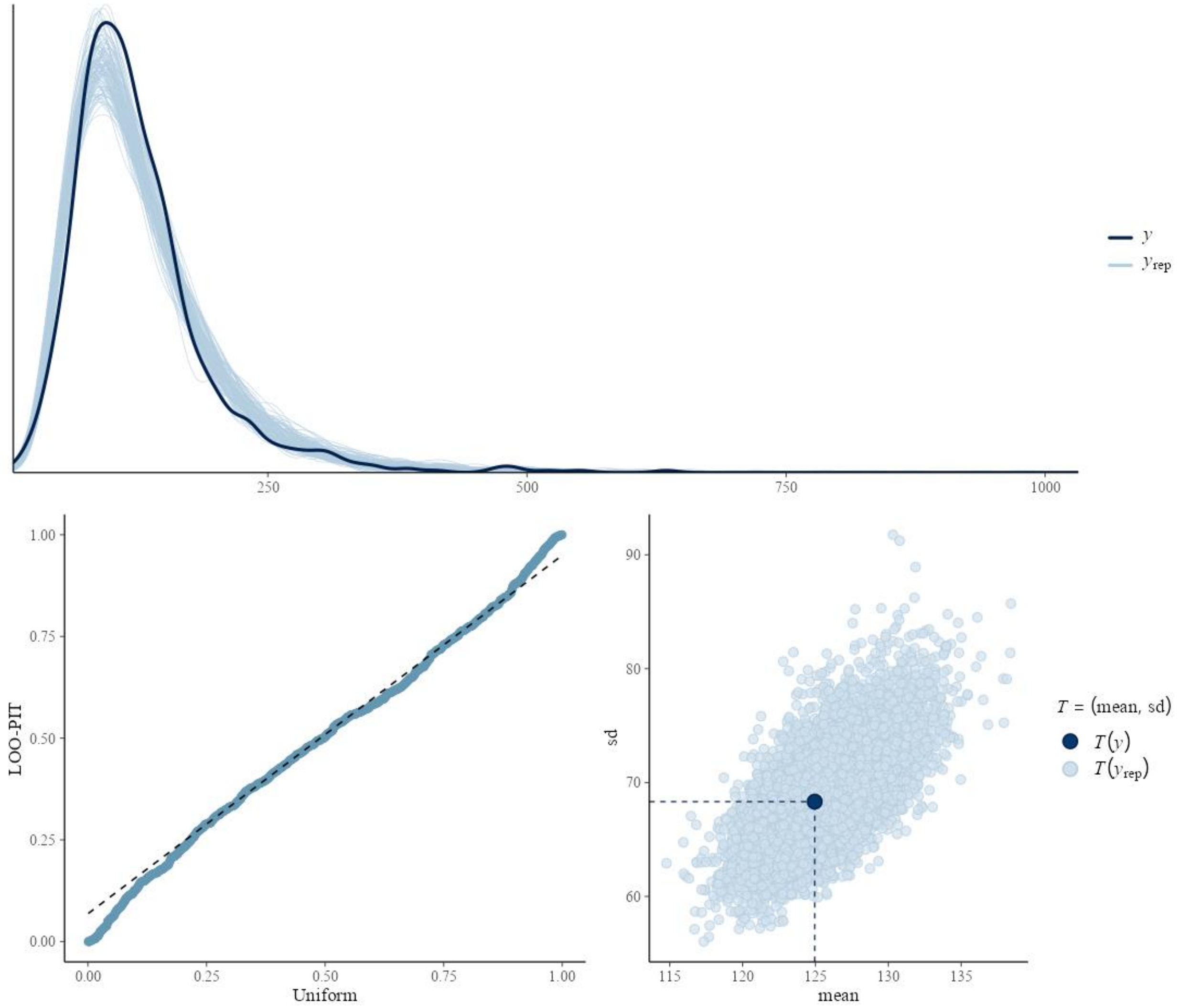

Supplementary Figure 7. Pairs plot to assess possible multicollinearity for the out-degree and out-strength model.

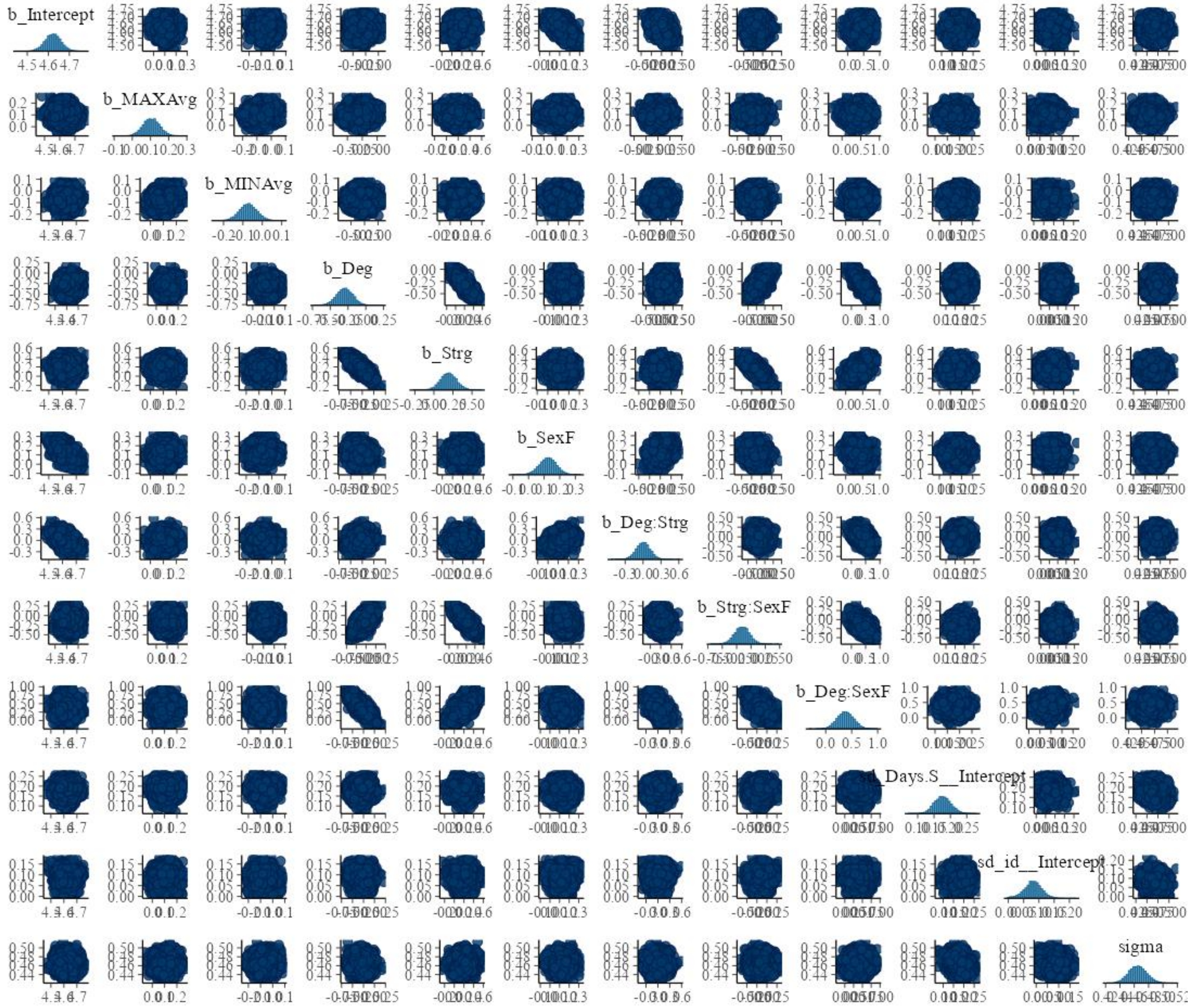

Supplementary Table 6. Variance inflation factors for the out-degree and out-strength model.

Term	VIF	VIF 95% CI	Increased SE	Tolerance	Tolerance 95% CI
MAXAvg	1.03	[1.01, 1.17]	1.02	0.97	[0.85, 0.99]
MINAvg	1.03	[1.00, 1.18]	1.01	0.97	[0.85, 1.00]
Out_Degree	8.02	[7.31, 8.82]	2.83	0.12	[0.11, 0.14]
Out_Strength	6.48	[5.92, 7.12]	2.55	0.15	[0.14, 0.17]
Sex	1.71	[1.60, 1.84]	1.31	0.59	[0.54, 0.63]
Out_Deg.:Out_Str.	1.98	[1.84, 2.14]	1.41	0.51	[0.47, 0.54]
Out_Deg:Sex	6.99	[6.37, 7.67]	2.64	0.14	[0.13, 0.16]
Out_Str:Sex	4.39	[4.02, 4.80]	2.09	0.23	[0.21, 0.25]
